# Association between the vertebrobasilar artery geometry and basilar artery plaques determined by high-resolution magnetic resonance imaging

**DOI:** 10.1186/s12868-021-00624-5

**Published:** 2021-03-25

**Authors:** Jinmei Zheng, Bin Sun, Ruolan Lin, Yongqi Teng, Xihai Zhao, Yunjing Xue

**Affiliations:** 1 Department of Radiology, Fujian Medical University Union Hospital, Fuzhou, 350001 Fujian China; 2 Department of Radiology, Changle District Hospital of Fuzhou, Fuzhou, 350299 Fujian China; 3 Center for Biomedical Imaging Research, Department of Biomedical Engineering, Tsinghua University School of Medicine, Beijing, 100084 China

**Keywords:** Atherosclerosis, Intracranial artery, Magnetic resonance imaging, Geometry, Vertebrobasilar

## Abstract

**Background:**

Atherosclerotic plaques are often present in regions of arteries with complicated flow patterns. Vascular morphology plays important role in hemodynamics. In this study, we investigated the relationship between the geometry of the vertebrobasilar artery system and presence of basilar artery (BA) plaque.

**Methods:**

We enrolled 290 patients with posterior circulation ischemic stroke. We distinguished four configurations of the vertebrobasilar artery: Walking, Tuning Fork, Lambda, and No Confluence. Patients were divided into multi-bending (≥ 3 bends) and oligo-bending (< 3 bends) VA groups. The diameter of the vertebral artery (VA) and the number of bends in the intracranial VA segment were assessed using three-dimensional time-of-flight magnetic resonance angiography. High-resolution magnetic resonance imaging was used to evaluate BA plaques. Logistic regression models were used to determine the relationship between the geometry type and BA plaque prevalence.

**Results:**

After adjusting for sex, age, body mass index ≥ 28, hypertension, and diabetes mellitus, the Walking, Lambda, and No Confluence geometries were associated with the presence of BA plaque (all *p* < 0.05). Patients with multi-bending VAs in both the Walking (20/28, 71.43% vs. 6/21, 28.57%, *p* = 0.003) and Lambda group (19/47, 40.43% vs. 21/97, 21.65%, *p* = 0.018) had more plaques compared to patients with oligo-bending VAs in these groups. In the Lambda group, the difference in diameter of bilateral VAs was larger in patients with BA plaques than that in patients without BA plaques (1.4 mm [IQR: 0.9–1.6 mm] vs. 0.9 mm [IQR: 0.6–1.3 mm], *p* < 0.001).

**Conclusions:**

The Walking, Lambda, and No Confluence geometry, ≥ 3 bends in the VAs, and a large diameter difference between bilateral VAs are associated with the presence of BA plaque.

**Supplementary Information:**

The online version contains supplementary material available at 10.1186/s12868-021-00624-5.

## Background

Intracranial atherosclerosis is an important cause of stroke worldwide [1] and accounts for almost 33–50% of ischemic strokes in Chinese population [2]. Posterior circulation strokes account for about 20–30% of all ischemic strokes [3, 4], and vertebrobasilar atherosclerosis is a common cause of posterior circulation ischemic strokes.

Identifying the morphology and composition of plaques has been shown to provide an incremental benefit over luminal stenosis alone when the aim is to define vulnerable lesions [5, 6] and predict subsequent cardiovascular ischemic events [7, 8], both in the coronary and carotid circulation. Several recent studies have confirmed the feasibility of using high-resolution magnetic resonance imaging (HRMRI) to evaluate intracranial arterial atherosclerosis [9, 10]. However, most of studies mainly focused on the relationship between plaque distribution, plaque enhancement, arterial remodeling patterns, intraplaque hemorrhage, and clinical ischemic events [11–14]. There is limited research that investigates the association between the vascular geometry and the plaque prevalence in posterior circulation using magnetic resonance imaging.

The risk factors of atherosclerosis is not only include systemic risk factors such as hypertension, smoking, hyperlipidemia, and diabetes mellitus, but also include focal vessel geometry [15]. The vertebrobasilar artery (VBA) system is unique in human anatomy in that two arteries (the left and right vertebral artery (VA)) merge into one artery (basilar artery (BA)). The diameters of the left and right VA differ in up to 50% of people [16], and their anatomical course is also different. Wake-Buck et al. [17] described the relationship between vertebrobasilar geometry and differences in hemodynamic distribution. Atherosclerotic plaques often develop in regions with low wall shear stress, such as the inner wall of a curved artery or the apex of a junction [18].

We hypothesized that different vertebrobasilar geometries with their specific hemodynamics will influence the presence of atherosclerotic plaques. This study aimed to explore the potential association between the presence of BA plaques and different vertebrobasilar geometries using HRMRI in vivo.

## Material and methods

### Patients

We consecutively recruited 303 patients who presented with a posterior circulation ischemic stroke in the Department of Neurology of our hospital between July 2017 and June 2018. All the recruited patients underwent HRMRI for intracranial arteries. Patients were included if they met the following criteria: (1) ischemic stroke or transient ischemic attack in the posterior circulation supplied by basilar artery presented with dizziness, unilateral limb weakness/ataxia, gait ataxia and so on; (2) quality of the HRMRI is sufficient for analysis, the quality of the image using a 1 to 3 scoring scale (1 = poor image quality: the presence of severe artifact impairing the diagnosis; 2 = image quality sufficient for diagnosis: mild or moderate artifact, not interfering with diagnosis; 3 = excellent image quality for highly confident diagnosis: no to minimal artifact). Our exclusion criteria were as follows: (1) nonatherosclerotic vasculopathy, such as dissection, arteritis or moyamoya disease; (2) evidence of a cardioembolic stroke (atrial fibrillation); (3) contraindications to MR imaging; (4) poor image quality due to severe motion artifact; (5) vascular geometry was not classified (see the Additional file 1: Figure S1). Based on these criteria, seven patients with severe motion artifacts, and six patients in whom vascular geometry could not be classified were excluded.

Eventually, 290 patients were enrolled in our study.

This study was conducted following the Declaration of Helsinki and was approved by the Ethics Committee of Union Medical College Hospital Affiliated to Fujian Medical University. Participants provided their written informed consent to participate in the research.

### Imaging protocol

Patients were scanned with a 3.0 T MR imaging system (Discovery MR750, General Electric Medical System, Milwaukee, WI, USA). The imaging protocols included T1-weighted imaging (T1WI), T2-weighted imaging (T2WI), diffusion-weighted imaging, three-dimensional time-of-flight magnetic resonance angiography (3D-TOF-MRA), and three-dimensional fast-spin-echo T1-weighted sequence (CUBE). The parameters of 3D-TOF-MRA were as follows: TR/TE = 19/3.5 ms, FOV = 180 mm × 180 mm, slice thickness = 1.2 mm, matrix = 288 × 288, bandwidth = 31.25 Hz, number of excitations = 1, the spatial resolution is 0.625mm × 0.625mm × 1.2 mm, acquisition time = 5 min 45 s. The imaging parameters of CUBE sequence were as follows: TR/TE = 600/16.5 ms, FOV = 180 mm × 180 mm, slice thickness = 0.8 mm, matrix = 288 × 288, bandwidth = 50 Hz, number of excitations = 2, ETL = 30, phase acceleration = 1.75, the spatial resolution is 0.625mm × 0.625mm × 0.8 mm, a slab-selective excitation was used, the coronal scanning coverage was from the basilar artery to the intracranial segment of vertebral arteries and the anterior circulation was not covered, acquisition time = 6 min 46 s. The coronal CUBE images were transferred to a local postprocessing station (Advantage Workstation, AW 4.5; General Electric Medical System, Milwaukee, WI, USA), and axial CUBE images were reformatted with sequential 1 mm intervals.

### Image analysis

**Fig. 1:**
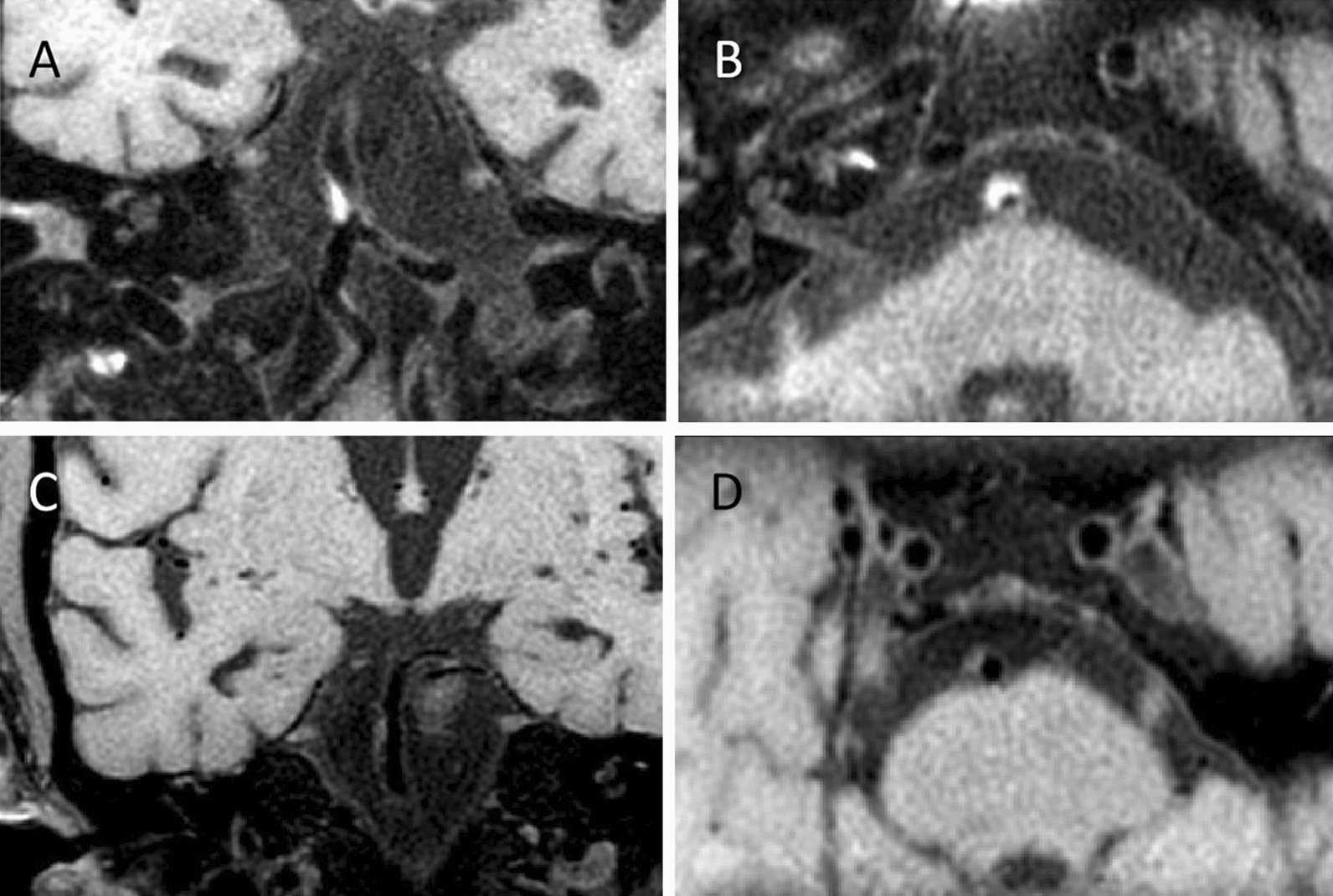
Example figures of plaques on 3D CUBE T1WI. An atherosclerotic plaque was present on coronal image (**a**) and corresponding reconstructed axial image (**b**) from a 79 years old male patient. Images C and D are from another male patient with 64 years old. An atherosclerotic plaque can be found on coronal image (**c**) and corresponding reconstructed axial image (**d**)

The atherosclerotic plaque was defined as an eccentric wall thickening, whereas the thinnest part was estimated to be < 50% of the thickest part by visual inspection on axial CUBE images [19] (Fig. 1). The presence or absence of BA plaque was identified by two experienced readers (Z.J. and X.Y.) blinded to MRA findings. The differences between the two observers were solved by consensus. To assess intra-observer reproducibility, CBUE images were reevaluated by one reviewer (Z.J.) one month later.

The diameter of the VA was measured on 3D-TOF-MRA. The diameter of each vessel was calculated as the average of the measurements made at three consecutive points, 3 mm apart, starting from the vertebrobasilar junction (for both VAs and the BA) (more details see Additional file 1: Figure S2) [16, 20]. The dominant VA was defined as: the diameter of the VA on the dominant side was larger than that of the contralateral VA (difference in diameter ≥ 0.3 mm) [16, 20].

**Fig. 2:**
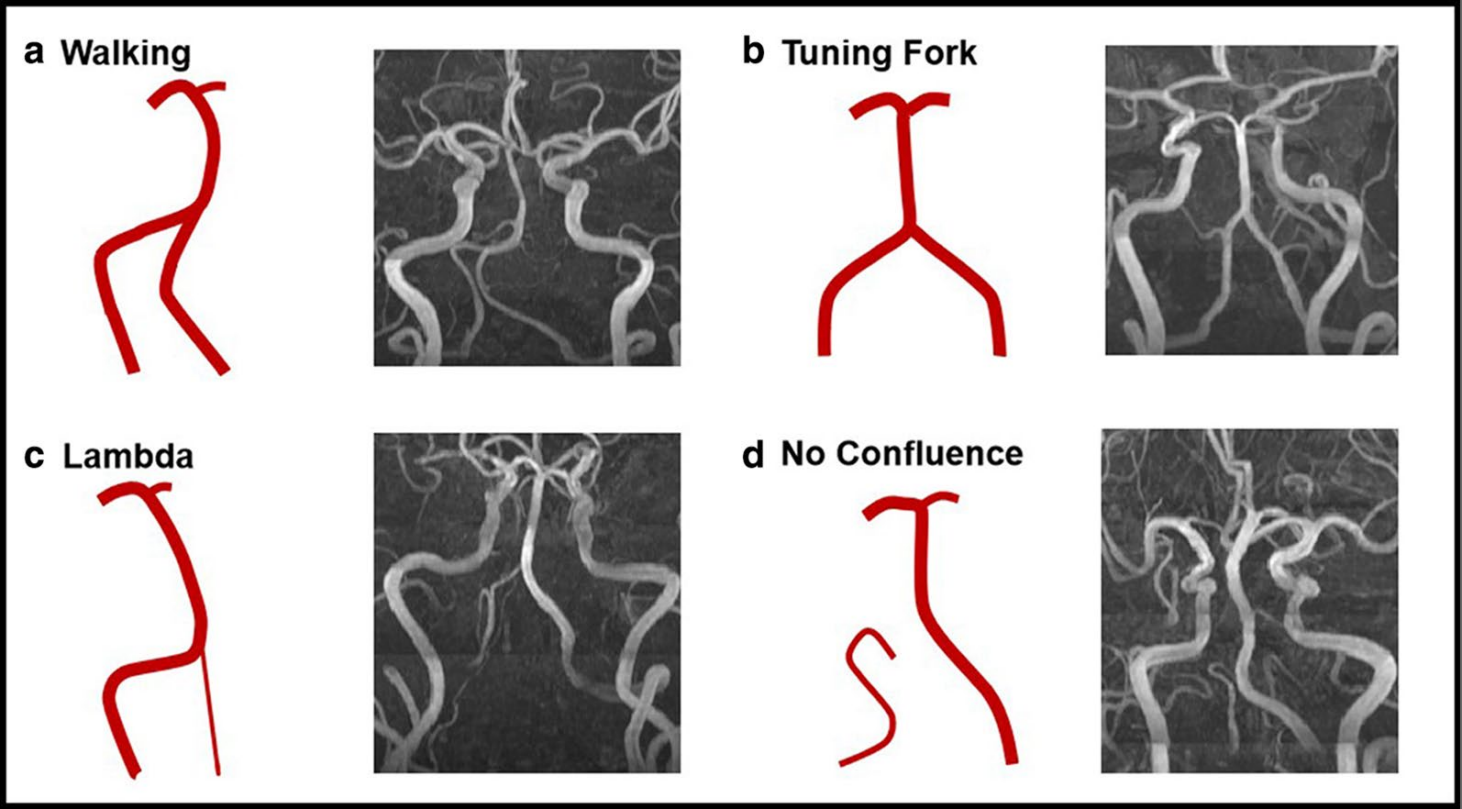
Examples for different geometry types of vertebrobasilar artery system. The vertebrobasilar artery geometry is qualitatively classified into four basic geometric configurations: Walking (**a**), Tuning Fork (**b**), Lambda (**c**), and No Confluence (**d**). In each panel, a schematic of the configuration is followed by anterior–posterior magnetic resonance angiography

On the 3D-TOF-MRA images, VBA geometry was qualitatively classified into four basic geometric configurations: Walking, Tuning Fork, Lambda, and No Confluence (Fig. 2). The Walking geometry is characterized by two VAs with a diameter difference of less than 0.3 mm that bend in the same direction before merging into the BA [16, 17]. The Tuning Fork is defined when two VAs have equal diameter (difference in diameter < 0.3 mm) and bend in opposite directions to form a symmetrical confluence from which the BA emerges [16, 17]. The Lambda geometry is defined as two VAs with a diameter difference of at least 0.3 mm that merge into the BA [16, 17]. In the No Confluence configuration, the VAs are not merged, of which one VA extends to be the BA, and the other VA feeds into other arteries, mostly the posterior inferior cerebellar artery.

**Fig. 3:**
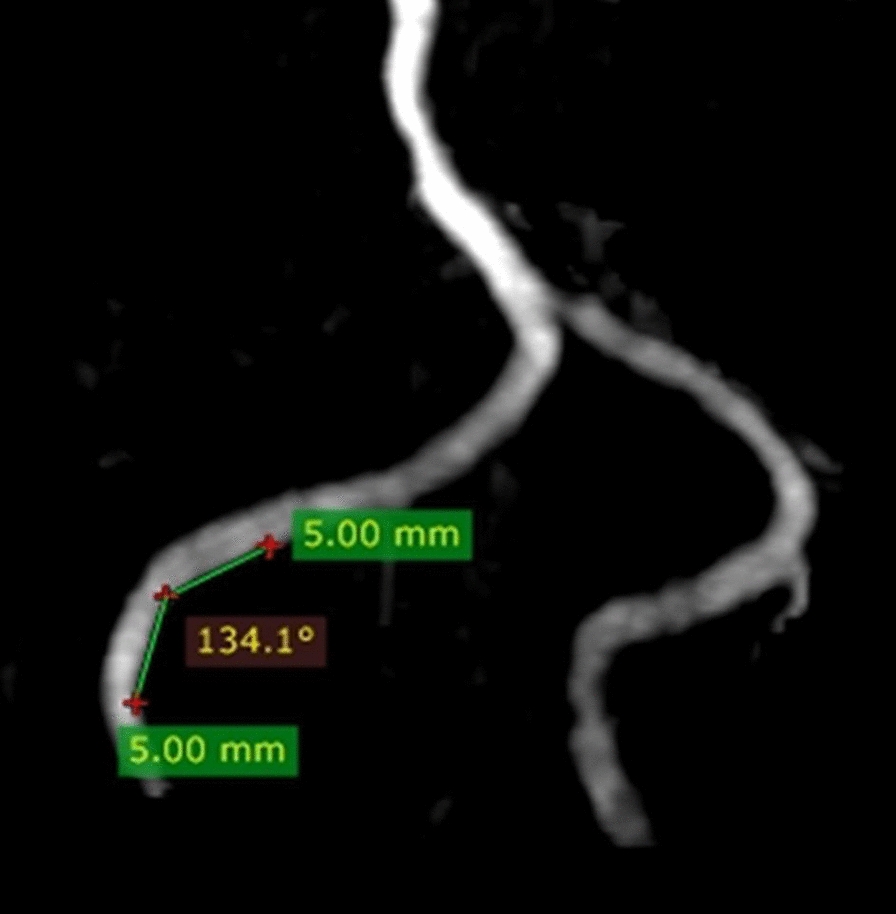
Schematic diagram of vascular curvature in vertebral artery. Two 5 mm long lines were drawn starting from the vertex of a vascular curve to both sides, the two lines intersect with the VA to form an angle. a vascular bend was identified If this angle was ≤ 150°

Vertebral artery is limited by posterior fossa bending mainly to left or right, the number of bends in the intracranial segments of the VAs was assessed on 3D-TOF-MRA images. Two 5 mm long lines were drawn starting from the vertex of a vascular curve to both sides, which intersect with the VA to form an angle. If this angle was ≤ 150°, it was defined as a vascular bend (Fig. 3). According to the total number of bends in the VAs’ intracranial segments, patients were divided into a multi-bending group (total number of bends ≥ 3) and an oligo-bending group (total number of bends < 3) (see Additional file 1: Figure S3). Vascular curvature was measured by the same two observers (Z.J. and X.Y.) one month later blinded to HRMRI findings. We took the consensus results from the measurements of two observers. To assess intra-observer reproducibility, vascular curvature was remeasured by one reviewer (Z.J.) one month later.

### Statistical analysis

Quantitative data with normal distribution were expressed as the mean ± standard deviation (SD), otherwise, were expressed as median (interquartile range, IQR), and qualitative data were expressed as percentages. The Shapiro–Wilk test for normality was used to investigate the distribution of data. Intra-observer or inter-observer variability for identification of BA plaque and vascular curvature measurements was performed using Cohen's kappa and intraclass correlation coefficient (ICC) analysis. The comparison of the BA plaque prevalence in the four different vascular geometries was performed using the chi-square test. Logistic regression models were use to determine the relationship between the geometry type and presence of BA plaque. The prevalence of BA plaque was compared between multi-bending and oligo-bending VAs using chi-square and Fisher exact test. In the case of the Lambda configuration, differences in the diameter between the right and left VAs for patients with and without BA plaques were compared with the Mann–Whitney U test, and a receiver operating characteristic curve analysis was used to determine the best cut-off value correlated with the presence of BA plaque. A *p*-value < 0.05 was considered to be statistically significant.

The statistical analysis was performed using IBM SPSS Statistics for Windows, version 19.0 (IBM Corp., Armonk, NY, USA).

## Results

The clinical characteristics of the study population are summarized in Table1.

**Table 1:** Clinical characteristics of the study population (n = 290)

	Mean ± SD or n(%)
Sex, male	174 (60.0)
Age, y	68.5 ± 10.3
Smoking	103 (35.52)
BMI ≥ 28	39 (13.45)
Hypertension	203 (70.00)
Diabetes mellitus	101 (34.83)
Hyperlipidemia	118 (40.69)
Coronary heart disease	36 (12.41)

Values are presented as mean ± standard deviation or number (%)

*BMI* body mass index

The mean age of the 290 patients was 68.5 ± 10.3 years, and 60% of which were males. A total of 86 patients (mean age, 70.2 ± 10.6 years) had BA plaques, and 204 patients (mean age, 67.7 ± 10.1 years) had no BA plaques. A body mass index ≥ 28, hypertension, and diabetes mellitus were more frequent in patients with BA plaques as compared to patients without BA plaques (all p < 0.05). The results on the comparison of clinical risk factors between patients with and without BA plaques is shown in Table 2. Of all 290 patients, 49 patients were in the Walking group, 73 patients were in the Tuning Fork group, 144 patients were in the Lambda group, and the last 24 patients were in the No Confluence group.

**Table 2:** Comparison of clinical risk factors between BA with and without plaque

	BA plaque ( +) (N = 86)	BA plaque (−) (N = 204)	*p*
Sex, male	50 (58.14)	124 (60.78)	0.675
Age, y	70.2 ± 10.6	67.7 ± 10.1	0.064
BMI ≥ 28	17 (19.77)	22 (10.78)	0.041
Smoking	29 (33.72)	74 (36.27)	0.678
Hypertension	69 (80.23)	134 (65.69)	0.014
DM	39 (45.35)	62 (30.39)	0.015
Hyperlipidemia	38 (44.19)	80 (39.22)	0.431
CHD	11 (12.79)	25 (12.25)	0.899

Values are presented as mean ± standard deviation or number (%)

*BMI* body mass index, *DM* diabetes mellitus, *CHD* coronary heart disease, *BA* basilar artery

The inter-observer (Kappa = 0.800) and intra-observer (Kappa = 0.898) agreement was excellent in identification of BA plaque. The inter-observer and intra-observer reproducibility for vascular curvature measurement were ICC = 0.905 (95% confidence interval [CI] 0.778–0.961) and ICC = 0.883 (95% CI 0.728–0.952), respectively.

### Correlation between VBA geometry and the presence of BA plaque

The prevalence of BA plaque was highest in patients with a Walking configuration (26/49, 53.06%) and lowest in patients with a Tuning Fork configuration (11/73, 15.07%). There was a significant difference in the prevalence of BA plaque among the four basic geometric configurations (*p* < 0.001, Table 3).

**Table 3:** Comparison of BA plaque prevalence in the four geometric configurations

VBA geometry	BA plaque ( +) (N = 86)	BA plaque (−) (N = 204)	*p*
			< 0.001
Walking(49)	26 (53.06)	23 (46.94)	
Tuning Fork(73)	11 (15.07)	62 (84.93)	
Lambda(144)	40 (27.78)	104 (72.22)	
No Confluence(24)	9 (37.50)	15 (62.50)	

Values are presented as number (%); BA, basilar artery; VBA, vertebrobasilar artery;

Walking vs. Tuning Fork, *p* ≤ 0.001; Walking vs. Lambda, *p* = 0.001; Walking vs. No Confluence, *p* = 0.211; Tuning Fork vs. Lambda, *p* = 0.037; Tuning Fork vs. No Confluence, *p* = 0.018; Lambda vs. No Confluence, *p* = 0.332; the significant level was adjusted to *p’*, *p’* = *p*/m, m = k (k-1)/2 + 1, k indicates the group number, and *p’* < 0.007 considered to be statistically significant

Table 4 shows the results on the logistic regression analysis for the association between VBA geometry and BA plaques. In the binary logistic regression analysis, Walking (odds ratio, 6.372; 95% confidence interval, 2.718–14.937; *p* < 0.001), Lambda (odds ratio, 2.168; 95% confidence interval, 1.037–4.533; *p* = 0.04), and No Confluence configuration (odds ratio, 3.382; 95% confidence interval, 1.188–9.625; *p* = 0.022) were found to be significantly associated with presence of BA plaque. After adjusting for age, sex, body mass index > 28, hypertension, and diabetes mellitus, these associations remained statistically significant (all *p* < 0.05; Table 4).

**Table 4:** Association between VBA Geometry and BA Plaque

VBA geometry	Model 0		Model 1	
	OR (95% CI)	*p* Value	OR (95% CI)	*p* Value
Tuning fork	Reference		Reference	
Walking	6.372 (2.718–14.937)	< 0.001	6.792 (2.805–16.447)	< 0.001
Lambda	2.168 (1.037–4.533)	0.04	2.420 (1.131–5.179)	0.023
No confluence	3.382 (1.188–9.625)	0.022	3.502 (1.179–10.406)	0.024

*OR* odds ratio, *CI* confidence interval, *BA* basilar artery, *VBA* vertebrobasilar artery

Model 0: no adjustment has been done; Model 1: adjust for sex, age, BMI ≥ 28, hypertension, and Diabetes mellitus

### Correlation between the number of vascular bends and the presence of BA plaque

In the Walking group, patients with multi-bending VAs had a higher prevalence of BA plaque than patients with oligo-bending VAs (20/28, 71.43% vs. 6/21, 28.57%, *p* = 0.003). In the Lambda group, patients with multi-bending VAs had a higher prevalence of BA plaque compared with patients with oligo-bending VAs (19/47, 40.43% vs. 21/97, 21.65%, *p* = 0.018). In the Tuning Fork group, none of the four patients with multi-bending VAs had BA plaques. In the No Confluence group, all patients had oligo-bending VAs (Table 5), and 9 patients (9/24, 37.5%) had BA plaques.

**Table 5:** Comparison of the prevalence of BA plaque between multi-bending and oligo-bending groups

	BA plaque ( +) (N = 86)	BA plaque (−) (N = 204)	*p*
Walking (49)			0.003
Multi-bending	20 (71.43%)	8 (28.57%)	
Oligo-bending	6 (28.57%)	15 (71.43%)	
Tuning Fork (73)			1.000
Multi-bending	0	4	
Oligo-bending	11 (15.94%)	58( 84.06%)	
Lambda (144)			0.018
Multi-bending	19 (40.43%)	28 (59.57%)	
Oligo-bending	21 (21.65%)	76 (78.35%)	
No confluence (24)			–
Multi-bending	0	0	
Oligo-bending	9	15	

Values are presented as number (%)

*BA* basilar artery, *VA* vertebral artery

### Correlation between difference in diameter between bilateral VAs and the presence of BA plaque

Twenty-four of the 290 patients (24/290, 8.3%) had a No Confluence configuration. In the 266 patients with either Walking, Tuning Fork, or Lambda, the average diameter of the left VA was 2.67 mm ± 0.64 mm and of the right VA was 2.47 mm ± 0.58 mm (*p* < 0.001), respectively. The diameters of the right and left VA were of equal size in 122 patients (122/266, 45.86%). Among the remaining 144 patients, left VA dominance was found in 95 (95/144, 65.97%) and right VA dominance was found in 49 (49/144, 34.03%) patients, respectively.

In the Lambda group, the mean diameter difference between bilateral VAs was 1.4 mm (0.9 mm -1.6 mm) in patients with BA plaques and 0.9 mm (0.6 mm-1.3 mm) in patients without BA plaques (*p* < 0.001), respectively. The receiver operating characteristic curve analysis identified a cut-off value for the difference in VAs diameter related to BA plaque formation of 1.35 mm.

## Discussion

This study investigated the relationship between VBA geometry and presence of BA plaque. We found that the prevalence of BA plaque was highest in the Walking geometry (26/49, 53.06%) and lowest in the Tuning Fork geometry (11/73, 15.07%). Moreover, the number of vascular bends in the intracranial segments of the VAs and the difference in diameter between the right and left VA were also associated with the presence of BA plaque.

Several previous HRMRI studies focused on the relationship between the vertebrobasilar artery geometry and BA plaque. Yu et al. [16] enrolled 84 patients with BA plaque and found that the BA plaques were evenly distributed in the vertebrobasilar arteries with Tuning Fork and Dominant-Lambda geometries. However, the BA plaques more frequently located in the dorsal or the lateral wall in patients with Hypoplasia-Lambda and Walking configurations. This study indicated that geometrical configurition of vertebrobasilar artery strongly influences the BA plaque distribution [16]. Compared to previous study, the presentt study has several strenghths: first, we not only focused on the Walking, Tuning Fork, and Lambda geometries, but also included the No Confluence configuration. We found that Walking, Lambda, and No Confluence were associated with the presence of BA plaque; second, we also discovered that multi-bending of VAs’ intracranial segments and the diameter difference between the right and left VAs also affected the presence of BA plaque; third, our study recruited 290 patients in the final analysis which increases the power of statistical analysis.

Several studies by Ravensbergen et al. [18, 21, 22] employed autopsy and a series of junction models, and demonstrated that vertebrobasilar geometry affects hemodynamics and atherosclerotic plaques frequently occur in regions with complex flow patterns and/or low wall shear stress. In a previous study, [17] high-field MRI was used in conjunction with computational fluid dynamics (CFD) modeling to investigate the hemodynamics of subject-specific confluence models (n = 5, two with Walking, two with Tuning Fork, and one with Dominant-Lambda geometry). This study showed that vertebrobasilar geometry strongly influences both the skewing of velocity profiles and wall shear stress distribution in the VBA system. In Walking geometry, the BA flow resulting from the merging of two VAs that bend in the same direction (right) makes the BA flow curving to the opposite direction (left). These chronic processes may induce a BA curvature. The shear stress is low at the inner wall of the BA curvature, and atherosclerotic plaques are prone to form in regions with low shear stress. Additionally, in the Walking geometry, the BA flow resulting from the VA flows swirling upward makes the flow distribution more complex, which can also induce plaque formation [17]. In the Tuning Fork geometry, the flows in the BA are roughly parallel, and the velocity profile peak in the BA is rather central [17], resulting in the low probability in formation of BA plaque in this geometric configuration.

In our study, the prevalence of BA plaque in the Lambda configuration was higher than that in the Tuning Fork configuration. Compared to Lambda patients without BA plaques, those with BA plaques had a larger difference in the diameters of bilateral VAs. In a study by Hong et al. [20] BA curvature was found to be associated with the diameter difference between bilateral VAs. The BA flow resulting from VAs with a diameter difference ≥ 0.3 mm makes the BA flow curved to the side of the weaker VA, and the chronic processes caused by the asymmetric VA flow induce greater curving of the BA wall, which may consequently cause atherogenesis.

We found that BA plaque prevalence in the No Confluence geometry was also higher than that in the Tuning Fork geometry. We hypothesize that BA flow coming from one VA also causes the BA flow curving to the wall opposite to the VA, and as a chronic process may consequently cause a curving of the BA wall. Subsequently, the deformation of the BA wall makes it prone to atherogenesis, which may lead to ischemic stroke in the posterior circulation. To date, there are no hemodynamic studies in patients with No Confluence geometry.

In our study, the prevalence of BA plaque was higher in patients with multi-bending VAs as compared to patients with oligo-bending VAs. We assumed that the flow patterns in the first group are more complex than that in the latter.

Our results have important clinical implications. First, we further classified VBA geometry based on the difference in diameter between the VAs and the VA course, which may help to improve the understanding of the vertebrobasilar system. Second, we demonstrated that the Walking, Lambda, and No Confluence geometry, multi-bending of the intracranial VA segment, and a large difference in the diameters of bilateral VAs are high-risk factors for BA atherosclerosis initiation. Therefore, people with these geometric factors should be intensively monitored for the progression or regression of BA plaque.

Our study has several limitations. First, we did not measure the hemodynamics and flow distribution in the four geometric configurations. The underlying mechanism of the vertebrobasilar artery geometry influencing the development of atherosclerotic plaques needs to be investigated from the hemodynamic aspect in future studies. Second, vascular curvature measurements were done manually from 2D images. Since 2D had limited orientation, the vascular curvature at other dimensions cannot be assessed and the processing of vascular curvature measurements may be affected as the effect of flow artifact on 3D-TOF-MRA. Third, coronal scanning was performed using the three-dimensional-CUBE sequence, and axial CUBE images were reconstructed. In determining presence of plaque, the wall thickness measurements from 3D multi-planar reformatting (MPR) could be affected by volume averaging artifacts as the image resolution is comparable to the artery wall. Fourth, this is a pilot study with a limited sample size. Future studies recruiting larger populations are warranted. Fifth, there may be some errors in categorization of the vertebral arteries because the cut-off value (i.e. 0.3 mm) of diameter difference was lower than the voxel size. However, such error is not expected to affect the main findings of this study. Finally, this study is an observational cross-sectional study which may be influenced by uncontrolled confounders.

## Conclusion

In conclusion, this study explored the correlation between vertebrobasilar geometry and the presence of BA plaque. The prevalence of BA plaque was highest in the Walking and lowest in the Tuning Fork configuration. Further, the extent of vascular bending in the intracranial VA segments and the diameter difference between bilateral VAs also were associated with the presence of BA plaque.

## Supplementary Information

**Additional file 1: Figure S1.** Figures of six patients whose vascular geometry were not classified. Bilateral vertebral arteries cross to the contralateral, left VA in the right and right VA in the left, to form the basilar artery in the patient 1–3. Bilateral vertebral arteries cross to the contralateral, but left VA in the left and right VA in the right, to form the basilar artery in the patient 4–5. Bilateral vertebral arteries curve medially before forming the basilar artery in the patient 6. **Figure S2.** Schematic diagram of the diameter measurement of the vertebral artery. The measurement was made at three consecutive points, 3 mm apart, starting from the vertebrobasilar junction, the diameter of the vertebral artery was calculated as the average of the three measurement values. For example: (2.73 mm + 2.63 mm + 2.53 mm)/3 = 2.63 mm. **Figure S3.** Schematic diagram of multi-bending and oligo-bending. (A). Four bends were identified in the bilateral vertebral arteries’ intracranial segments, the patient was classified into the multi-bending group (total number of bends ≥ 3). (B). Not bend was identified in the intracranial segments of the vertebral arteries, the patient was assigned to the oligo-bending group (total number of bends < 3).

## Data Availability

The datasets used and/or analyzed during the current study are available from the corresponding author on reasonable request.

## Abbreviations

BA: Basilar artery
VA: Vertebral artery
VBA: Vertebrobasilar artery
3D-TOF-MRA: Three-dimensional time-of-flight magnetic resonance angiography
HRMRI: High-resolution magnetic resonance imaging

## References

[1] Gorelick PB, Wong KS, Bae HJ, Pandey DK (2008). Large artery intracranial occlusive disease: a large worldwide burden but a relatively neglected frontier. Stroke.

[2] Wong LK (2006). Global burden of intracranial atherosclerosis. Int J Stroke.

[3] Amin-Hanjani S, Pandey DK, Rose-Finnell L, Du X, Richardson D, Thulborn KR, Elkind MS, Zipfel GJ, Liebeskind DS, Silver FL, Kasner SE, Aletich VA, Caplan LR, Derdeyn CP, Gorelick PB, Charbel FT (2016). Vertebrobasilar flow evaluation and risk of transient ischemic attack and stroke study group. Effect of hemodynamics on stroke risk in symptomatic atherosclerotic vertebrobasilar occlusive disease. JAMA Neurol.

[4] Nouh A, Remke J, Ruland S (2014). Ischemic posterior circulation stroke: a review of anatomy, clinical presentations, diagnosis, and current management. Front Neurol.

[5] Underhill HR, Hatsukami TS, Fayad ZA, Fuster V, Yuan C (2010). MRI of carotid atherosclerosis: clinical implications and future directions. Nat Rev Cardiol.

[6] Garcia-Garcia HM, Costa MA, Serruys PW (2010). Imaging of coronary atherosclerosis: intravascular ultrasound. Eur Heart J.

[7] Stone GW, Maehara A, Lansky AJ, de Bruyne B, Cristea E, Mintz GS, Mehran R, McPherson J, Farhat N, Marso SP, Parise H, Templin B, White R, Zhang Z, Serruys PW, PROSPECT Investigators (2011). A prospective natural-history study of coronary atherosclerosis. N Engl J Med..

[8] Saam T, Hetterich H, Hoffmann V, Yuan C, Dichgans M, Poppert H, Koeppel T, Hoffmann U, Reiser MF, Bamberg F (2013). Meta-analysis and systematic review of the predictive value of carotid plaque hemorrhage on cerebrovascular events by magnetic resonance imaging. J Am Coll Cardiol.

[9] Ryu CW, Jahng GH, Kim EJ, Choi WS, Yang DM (2009). High resolution wall and lumen MRI of the middle cerebral arteries at 3 tesla. Cerebrovasc Dis.

[10] Li ML, Xu WH, Song L, Feng F, You H, Ni J, Gao S, Cui LY, Jin ZY (2009). Atherosclerosis of middle cerebral artery: evaluation with high-resolution MR imaging at 3T. Atherosclerosis.

[11] Yu JH, Kwak HS, Chung GH, Hwang SB, Park MS, Park SH (2015). Association of intraplaque hemorrhage and acute infarction in patients with basilar artery plaque. Stroke.

[12] Wang W, Yang Q, Li D, Fan Z, Bi X, Du X, Wu F, Wu Y, Li K (2017). Incremental value of plaque enhancement in patients with moderate or severe basilar artery stenosis: 3.0 T high-resolution magnetic resonance study. Biomed Res Int.

[13] Ma N, Jiang WJ, Lou X, Ma L, Du B, Cai JF, Zhao TQ (2010). Arterial remodeling of advanced basilar atherosclerosis: a 3-tesla MRI study. Neurology.

[14] Yu J, Li ML, Xu YY, Wu SW, Lou M, Mu XT, Feng F, Gao S, Xu WH (2017). Plaque distribution of low-grade basilar artery atherosclerosis and its clinical relevance. BMC Neurol.

[15] Malek AM, Alper SL, Izumo S (1999). Hemodynamic shear stress and its role in atherosclerosis. JAMA.

[16] Yu J, Zhang S, Li ML, Ma Y, Dong YR, Lou M, Feng F, Gao S, Wu SW, Xu WH (2018). Relationship between the geometry patterns of vertebrobasilar artery and atherosclerosis. BMC Neurol.

[17] Wake-Buck AK, Gatenby JC, Gore JC (2012). Hemodynamic characteristics of the vertebrobasilar system analyzed using MRI-based models. PLoS ONE.

[18] Ravensbergen J, Ravensbergen JW, Krijger JK, Hillen B, Hoogstraten HW (1998). Localizing role of hemodynamics in atherosclerosis in several human vertebrobasilar junction geometries. Arterioscler Thromb Vasc Biol.

[19] Xu WH, Li ML, Gao S, Ni J, Zhou LX, Yao M, Peng B, Feng F, Jin ZY, Cui LY (2011). Plaque distribution of stenotic middle cerebral artery and its clinical relevance. Stroke.

[20] Hong JM, Chung CS, Bang OY, Yong SW, Joo IS, Huh K (2009). Vertebral artery dominance contributes to basilar artery curvature and peri-vertebrobasilar junctional infarcts. J Neurol Neurosurg Psychiatry.

[21] Ravensbergen J, Krijger JK, Verdaasdonk AL, Hillen B, Hoogstraten HW (1997). The influence of the blunting of the apex on the flow in a vertebro-basilar junction model. J Biomech Eng.

[22] Ravensbergen J, Krijger JK, Hillen B, Hoogstraten HW (1996). The influence of the angle of confluence on the flow in a vertebro-basilar junction Model. J Biomech.

