# Supplementary material for: Association between the vertebrobasilar artery geometry and basilar artery plaques determined by high-resolution magnetic resonance imaging
Source: BMC Neurosci. 2021 Mar 25;22:20. doi: 10.1186/s12868-021-00624-5 (PMC7992992; doi:10.1186/s12868-021-00624-5)
Supplement: Supplementary file 1 — Additional file 1: Figure S1. Figures of six patients whose vascular geometry were not classified. Bilateral vertebral arteries cross to the contralateral, left VA in the right and right VA in the left, to form the basilar artery in the patient 1–3. Bilateral vertebral arteries cross to the contralateral, but left VA in the left and right VA in the right, to form the basilar artery in the patient 4–5. Bilateral vertebral arteries curve medially before forming the basilar artery in the patient 6. Figure S2. Schematic diagram of the diameter measurement of the vertebral artery. The measurement was made at three consecutive points, 3 mm apart, starting from the vertebrobasilar junction, the diameter of the vertebral artery was calculated as the average of the three measurement values. For example: (2.73 mm + 2.63 mm + 2.53 mm)/3 = 2.63 mm. Figure S3. Schematic diagram of multi-bending and oligo-bending. (A). Four bends were identified in the bilateral vertebral arteries’ intracranial segments, the patient was classified into the multi-bending group (total number of bends ≥ 3). (B). Not bend was identified in the intracranial segments of the vertebral arteries, the patient was assigned to the oligo-bending group (total number of bends < 3). [file 12868_2021_624_MOESM1_ESM.pdf]

**Title:**

# Association between the vertebrobasilar artery geometry and basilar artery plaques determined by high-resolution magnetic resonance imaging

**Authors' name:**

Jinmei Zheng[1], Bin Sun[1], Ruolan Lin[1], Yongqi Teng[2], Xihai Zhao[3], Yunjing Xue[1]

**Authors' affiliations:**

1. Department of Radiology, Fujian Medical University Union Hospital, Fuzhou, 350001, Fujian, China

2. Department of Radiology, Changle District Hospital of Fuzhou, Fuzhou, 350299, Fujian, China

3. Center for Biomedical Imaging Research, Department of Biomedical Engineering, Tsinghua University School of Medicine, Beijing, 100084, China

**Corresponding author:**

Correspondence to Dr. Yunjing Xue

Yunjing Xue, Fujian Medical University Union Hospital, Fujian, 350001, China. Tel: 86-0591-83333151. Fax: 86-0591-83333151. Email: xueyunjing@126.com

**Figure S1:**

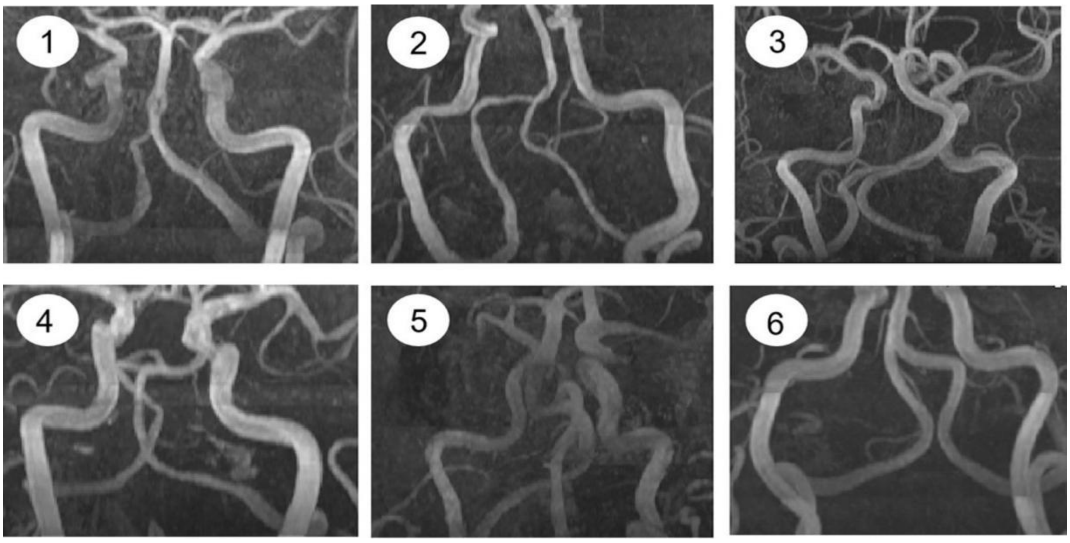


Figure S1：Figures of six patients whose vascular geometry were not classified. Bilateral vertebral arteries cross to the contralateral, left VA in the right and right VA in the left, to form the basilar artery in the patient 1-3. Bilateral vertebral arteries cross to the contralateral, but left VA in the left and right VA in the right, to form the basilar artery in the patient 4-5. Bilateral vertebral arteries curve medially before forming the basilar artery in the patient 6.

**Figure S2:**

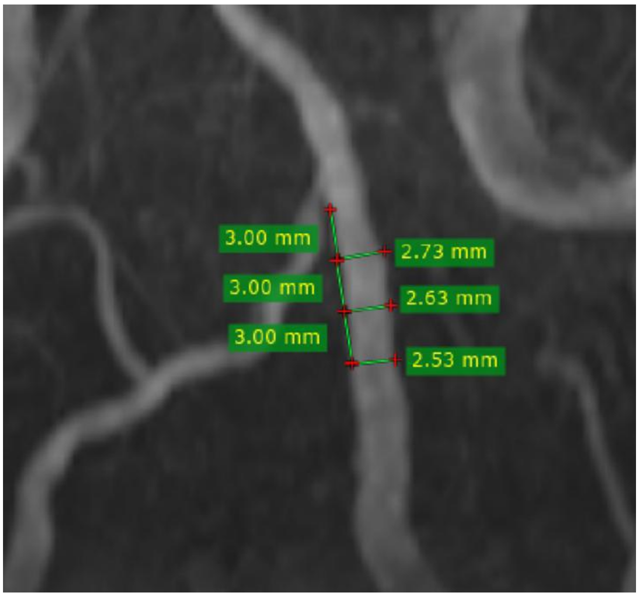


Figure S2: Schematic diagram of the diameter measurement of the vertebral artery. The measurement was made at three consecutive points, 3 mm apart, starting from the vertebrobasilar junction, the diameter of the vertebral artery was calculated as the average of the three measurement values. For example: (2.73 mm + 2.63 mm + 2.53 mm)/3 = 2.63 mm.

**Figure S3:**

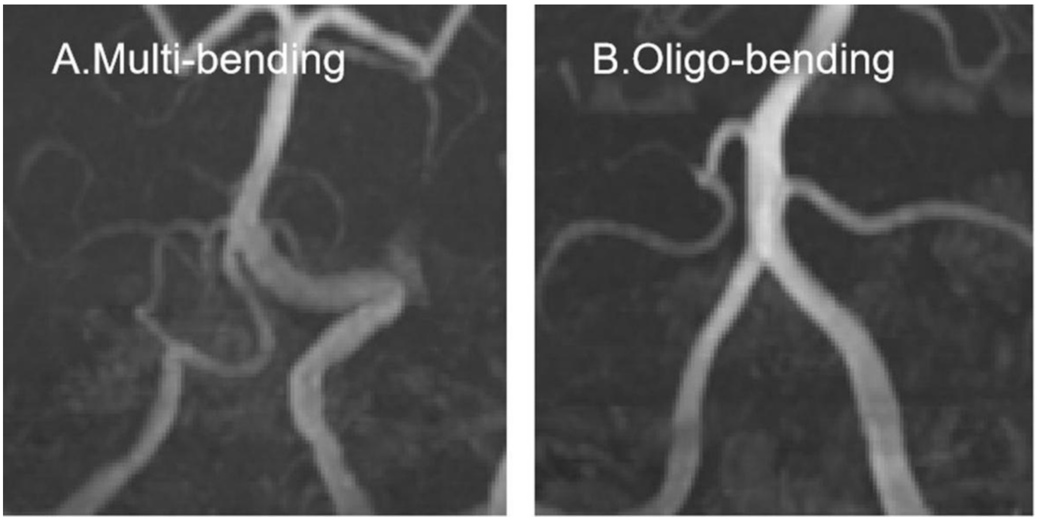


Figure S3: Schematic diagram of multi-bending and oligo-bending.

(A). Four bends were identified in the bilateral vertebral arteries' intracranial segments, the patient was classified into the multi-bending group (total number of bends ≥ 3). (B). Not bend was identified in the intracranial segments of the vertebral arteries, the patient was assigned to the oligo-bending group (total number of bends < 3).
